# Loci Contributing to Boric Acid Toxicity in Two Reference Populations of *Drosophila melanogaster*

**DOI:** 10.1534/g3.117.041418

**Published:** 2017-06-05

**Authors:** Michael A. Najarro, Jennifer L. Hackett, Stuart J. Macdonald

**Affiliations:** * Department of Molecular Biosciences, University of Kansas, Lawrence, Kansas 66045; † Center for Computational Biology, University of Kansas, Lawrence, Kansas 66047

**Keywords:** complex traits, MPP, RNAi, quantitative trait loci, xenobiotics, Multi-parent Advanced Generation Inter-Cross (MAGIC), multiparental populations

## Abstract

Populations maintain considerable segregating variation in the response to toxic, xenobiotic compounds. To identify variants associated with resistance to boric acid, a commonly-used household insecticide with a poorly understood mechanism of action, we assayed thousands of individuals from hundreds of strains. Using the *Drosophila* Synthetic Population Resource (DSPR), a multi-parental population (MPP) of inbred genotypes, we mapped six QTL to short genomic regions containing few protein-coding genes (3–188), allowing us to identify plausible candidate genes underlying resistance to boric acid toxicity. One interval contains multiple genes from the cytochrome P450 family, and we show that ubiquitous RNAi of one of these genes, *Cyp9b2*, markedly reduces resistance to the toxin. Resistance to boric acid is positively correlated with caffeine resistance. The two phenotypes additionally share a pair of QTL, potentially suggesting a degree of pleiotropy in the genetic control of resistance to these two distinct xenobiotics. Finally, we screened the *Drosophila* Genetic Reference Panel (DGRP) in an attempt to identify sequence variants within mapped QTL that are associated with boric acid resistance. The approach was largely unsuccessful, with only one QTL showing any associations at QTL-specific 20% False Discovery Rate (FDR) thresholds. Nonetheless, these associations point to a potential candidate gene that can be targeted in future validation efforts. Although the mapping data resulting from the two reference populations do not clearly overlap, our work provides a starting point for further genetic dissection of the processes underlying boric acid toxicity in insects.

A major goal of quantitative genetics is to identify and characterize sequence variants contributing to the extensive interindividual variation observed for medically- and evolutionarily-relevant traits. Genetic mapping studies can implicate novel pathways, genes, and variants in the genetic control of disease risk and phenotypic variation. More broadly, similar investigations across multiple traits and systems have the potential to provide answers to fundamental questions about complex traits; for example, are causative alleles generally rare or at intermediate frequency, and do they segregate for a series of alleles that impact phenotype? Any general patterns uncovered can lead to predictions about the evolutionary forces acting on functional genetic variation, and aid in the development of novel, more powerful methods for the dissection of trait variation.

Essentially, any trait with a significant fraction of heritable variation is open to genetic dissection by linkage- or association-based mapping techniques. However, work in both model systems (*e.g.*, Valdar *et al.* 2006b; Rat Genome Sequencing and Mapping Consortium *et al.* 2013) and in humans (*e.g.*, Yang *et al.* 2010) indicates that, on average, causative loci confer only fairly subtle effects on phenotype. Thus, mapping studies must involve large numbers of individuals to find variants with realistic effect sizes, meaning that traits that can be assayed in a high-throughput fashion are more tractable. Toxic compounds, or xenobiotics, are easily delivered to flies by feeding, facilitating powerful mapping studies using hundreds of strains and tens of thousands of individuals (*e.g.*, Najarro *et al.* 2015). Since xenobiotic resistance can also exhibit relatively high heritability in *Drosophila melanogaster* (Carrillo and Gibson 2002; Marriage *et al.* 2014; Najarro *et al.* 2015), genetic dissection of many such traits may help elucidate general properties of complex trait variation.

All animals face considerable pressure from exposure to xenobiotics in their diet and environment, from toxins produced by plants as protection against herbivory (Glendinning 2007; Mithofer and Boland 2012) to agricultural pesticides (Perry *et al.* 2011). As a result, animal genomes possess a diverse array of genes responsible for the metabolism and excretion of toxins. These genes, including cytochrome P450 monooxygenases (P450s), glutathione-S-transferases, UDP-glucuronosyltransferases, and ABC transporters, coordinate to form a sophisticated detoxification system (Xu *et al.* 2005; Li *et al.* 2007). These gene families are strong candidates to harbor segregating variation contributing to differences in xenobiotic resistance among individuals. Indeed, one of the best examples of xenobiotic metabolism in insects is the series of structural changes at the P450 gene *Cyp6g1* that are largely responsible for resistance to the insecticide DDT in populations of *D. melanogaster* (Daborn *et al.* 2002; Chung *et al.* 2007; Schmidt *et al.* 2010).

Unbiased, genome-wide mapping studies for resistance to caffeine (Najarro *et al.* 2015) and nicotine (Marriage *et al.* 2014) have identified QTL collectively explaining significant fractions of trait heritability, and have implicated known detoxification genes in the genetic control of resistance. Here, we complement this work by dissecting the genetic basis of resistance to boric acid, a common household insecticide. Boric acid has been shown to be effective against various ant species (Klotz and Moss 1996; Klotz *et al.* 1996), and the larvae of both honeybees (da Silva Cruz *et al.* 2010) and wax moths (Hyrsl *et al.* 2007; Buyukguzel *et al.* 2013). Boric acid administration has morphological effects on the midgut and malpighian tubules in leaf-cutting ants (Sumida *et al.* 2010), leads to abnormalities in midgut cells in Argentine ants (Klotz *et al.* 2002) and honeybee larvae (da Silva Cruz *et al.* 2010), and promotes irregularities in both midgut tissue and the fat body in wax moths (Buyukguzel *et al.* 2013). Nonetheless, the physiological mechanisms responsible for the mortality associated with boric acid intake are not clear. Genetic dissection of variation in resistance in the *D. melanogaster* model system has the potential to provide insight into the genes involved in boric acid toxicity.

A powerful strategy to identify causative loci is to employ a series of recombinant genotypes derived from a small number of founding haplotypes. So-called MPPs have been developed for the major animal model systems, including the Collaborative Cross (Churchill *et al.* 2004; Threadgill and Churchill 2012) and the Diversity Outbred (DO, Churchill *et al.* 2012; Svenson *et al.* 2012) mouse populations, the NIH-HS rat heterogeneous stock (Rat Genome Sequencing and Mapping Consortium *et al.* 2013), and the DSPR (King *et al.* 2012b). These populations facilitate statistically powerful QTL mapping (Valdar *et al.* 2006a; King *et al.* 2012a; Gatti *et al.* 2014), are capable of resolving QTL to shorter intervals than is generally possible with an F_2_ mapping design due to the multiple generations of intercrossing typically employed, and as a result of being founded from multiple strains allow the characterization of allelic series at functional loci. In addition, stable reference populations such as the DSPR allow multiple traits, including “molecular phenotypes” such as chromatin accessibility and gene expression, to be measured on the same set of genotypes. In so doing, reference populations facilitate examinations of pleiotropy, as well as enabling characterization of the functional, molecular consequences of sequence-level variation.

In this study, we describe the genetic dissection of boric acid resistance in both the DSPR MPP and in a second reference mapping population, the DGRP (Mackay *et al.* 2012), a series of resequenced inbred strains allowing genome-wide association testing. We address three basic questions. First, can we identify QTL and uncover plausible candidate genes contributing to resistance to boric acid in flies? By working with *D. melanogaster* we can ultimately leverage the power of this model system to increase the depth of our mechanistic understanding of boric acid toxicity in insects. Second, can we find evidence for pleiotropic resistance loci? We previously used a nearly identical phenotyping assay to examine caffeine resistance in the DSPR (Najarro *et al.* 2015). Correlation between caffeine and boric acid resistance measured in the same set of genotypes, as well as any overlap in the QTL mapped, would imply some level of pleiotropy. Third, can we identify sequence variants contributing to trait variation by combining high-resolution QTL mapping with GWAS (genome-wide association study) data? GWAS have low power due to the strict correction for multiple tests that must be applied to avoid false positive associations, and one approach to mitigate this power deficit is to test associations only within those narrow intervals implicated by QTL mapping.

## Materials and Methods

### Phenotyping pipeline

Our measure of resistance to boric acid is taken as the lifespan (in hours) of mated females exposed to media supplemented with 1.5% boric acid (BH_3_O_3_, BP168; Fisher Scientific). This concentration was arrived at following a series of *ad hoc* experiments testing a number of *D. melanogaster* strains against a range of boric acid concentrations. We assayed resistance for all genotypes following an identical pipeline to that described previously for caffeine resistance (Najarro *et al.* 2015). Briefly, parental flies were permitted to lay eggs for up to 2 d, clearing when needed to maintain roughly similar egg density across experimental vials. After 8 d any adult progeny were cleared, and 2 d later female flies were collected under CO_2_ anesthesia. By this point all vials contained many flies of both sexes, so we assume that females were generally mated. Experimental females were kept in single-sex groups of ∼20 flies on regular media for 1 d to recover from anesthesia. Subsequently, test flies were aspirated individually, and without anesthesia into narrow polycarbonate tubes containing a small quantity of cornmeal-yeast-dextrose media supplemented with 1.5% boric acid. Media was made 24 hr prior to the start of the assay, and boric acid was added to the molten media at ∼50° to minimize any heat-induced degradation. Tubes were inserted into *Drosophila* Activity Monitoring System units (DAM2; TriKinetics), and the activity of each animal was recorded every minute for 6 d using the DAMSystem3 data collection software (TriKinetics). Fly death was indicated by a permanent cessation of activity.

All experimental individuals were reared and assayed at 25° and 50% relative humidity on a 12 hr light/12 hr dark cycle.

### QTL mapping

We employed the DSPR MPP to find QTL contributing to boric acid resistance. The DSPR consists of two panels of Recombinant Inbred Lines (RILs) (pA and pB), each derived from a pair of replicate highly-recombinant synthetic populations (pA1 and pA2, and pB1 and pB2) initiated with a different set of eight inbred founder strains (pA RILs founded by strains A1–A7 and AB8, and pB RILs founded by strains B1–B7 and AB8). Detailed descriptions of RIL construction, founder resequencing, and RIL genotyping-by-sequencing are provided in King *et al.* (2012b). For this study, we screened through 835 pA and 855 pB RILs, measuring boric acid resistance for eight replicate individuals per RIL (Supplemental Material, File S1). The 1690 RILs were tested in 13 batches, with each RIL being tested in a single batch.

**Figure 1:**
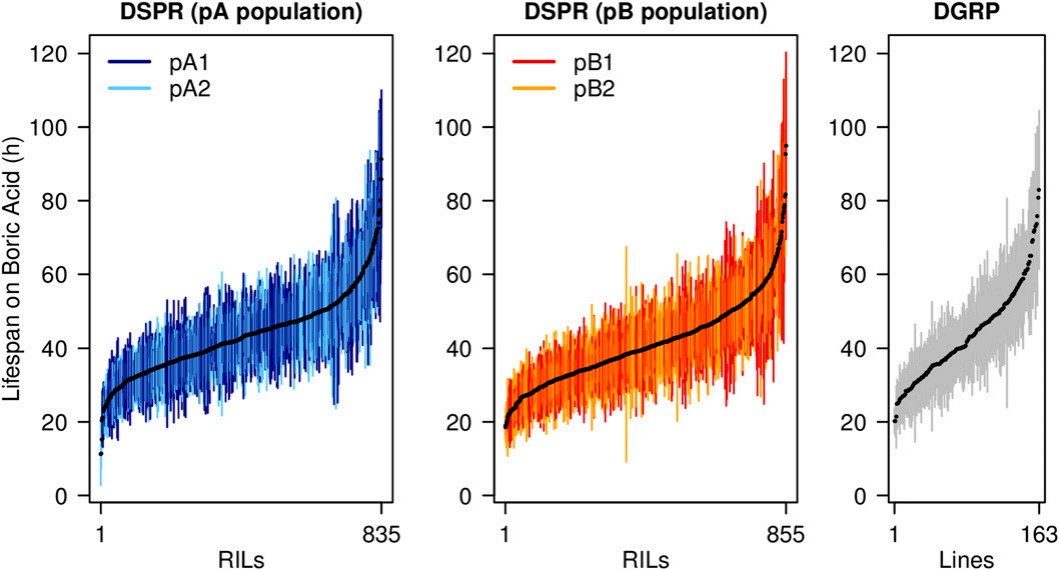
Phenotype distributions in the reference populations. We measured lifespan on media supplemented with 1.5% boric acid for 1690 RILs from the DSPR and 163 inbred lines from the DGRP. We scored eight females from each DSPR RIL and 16 females from each DGRP strain. Each plot shows the mean (filled circles) and 1-SD (vertical lines) for each genotype. RILs derived from each of the DSPR subpopulations (pA1, pA2, pB1, pB2) are shown in different colors; No significant differences in RIL mean phenotype were found between subpopulations (Welch’s *t*-tests, *P* > 0.2). DGRP, *Drosophila* Genetic Reference Panel; DSPR, *Drosophila* Synthetic Population Resource; RILs, Recombinant Inbred Lines.

QTL were mapped by providing RIL means to the R package *DSPRqtl* (version 2.0-5), that depends on the data packages *DSPRqtlDataA* and *DSPRqtlDataB* (version 2.0-1) to provide the RIL genotypes. Packages are available at FlyRILs.org and GitHub (github.com/egking/DSPRqtl), and genotypes can be obtained via FlyRILs.org and Dryad (dx.doi.org/10.5061/dryad.r5v40). Methods for QTL mapping in the DSPR are thoroughly described in King *et al.* (2012a,b). Briefly, at each position in each RIL we assign an additive probability that the genetic material is derived from each of the eight possible founders, then regress mean RIL phenotype on these eight probabilities using the model $y=\mu +\sum_{i=1}^{7}\beta_{i}G_{i},$ where μ is the grand mean, *G_i_* are the genotype probabilities, and *β_i_* are the additive genetic effects of each haplotype. The resulting *F*-statistic is then converted to a LOD score (Broman and Sen 2009). The pA and pB panels were analyzed separately, ignoring subpopulation since there was no significant difference in the average phenotype of RILs from each subpopulation (Figure 1; pA1 *vs.* pA2, Welch’s *t*-test, *P* = 0.34; pB1 *vs.* pB2, *P* = 0.21). Genome-wide 5% significance thresholds for calling QTL were estimated via 1000 permutations of the data (following Churchill and Doerge 1994), and we used 2-LOD support intervals to estimate the true positions of causative sites (King *et al.* 2012a).

### Association mapping

We employed strains from the DGRP (Mackay *et al.* 2012; Huang *et al.* 2014) to identify sites associated with variation in boric acid resistance. DGRP strains were purchased from the Bloomington *Drosophila* Stock Center (BDSC). Following the pipeline described above, we collected phenotypes for 16 replicate individuals from each of 163 inbred DGRP strains (File S1), assaying the strains in four batches. Strain means were subsequently submitted to the DGRP2 analytical engine (dgrp2.gnets.ncsu.edu) for GWAS analysis. Huang *et al.* (2014) provide thorough detail of the methodology employed. Briefly, the pipeline involves first adjusting line means to account for variation across lines in both *Wolbachia* infection status and the presence of large chromosomal inversions. Next, all common (minor allele frequency > 0.05) variants are tested for an association with phenotype using a linear mixed model that accounts for variation in relatedness among strains, *y* = ***S****b* + ***Z****u* + *e*, where ***S*** is the design matrix for fixed SNP effect *b*, ***Z*** is the incidence matrix for the random polygenic effect *u*, and *e* is the residual (Huang *et al.* 2014). We sought to identify variants significantly associated with phenotype by using a Bonferroni correction for multiple tests, and by using a FDR (Benjamini and Hochberg 1995) as implemented by the *p.adjust* function in R (r-project.org).

### Heritability of the phenotype

We calculated estimates of the broad-sense heritability of boric acid resistance for both panels of the DSPR and for the DGRP. Genetic and phenotypic variance components were calculated via the *lme* and *VarCorr* functions from the *nlme* R package (Pinheiro *et al.* 2011), using the linear mixed model *Y_ij_* = μ + *g_i_* + *ε_ij_*, where *Y_ij_* is the *j*th observation of the *i*th RIL, μ is the grand mean, *g_i_* is the random effect of RIL, and *ε_ij_* is the error term.

### Functional testing of candidate genes via RNAi

We used the binary Gal4-UAS RNAi system to test a pair of candidate detoxification genes, *Cyp6a2* and *Cyp9b2*, residing under DSPR QTL pB.Q1 for any effects on phenotype. We drove Gal4 in all cells at all timepoints using *Act5C*-GAL4 (from BDSC stock 25374). Transgenic RNAi Project (TRiP, Perkins *et al.* 2015) UAS-RNAi and co-isogenic control strains were obtained from the BDSC, specifically stock numbers 35786 (UAS*-GFP* control), 35788 (UAS*-Luciferase* control), 62996 (UAS-*Cyp9b2-RNAi*), and 64008 (UAS-*Cyp6a2-RNAi*). We generated RNAi knockdown/control genotypes by crossing five males from the Gal4 strain to 10 virgin females from each UAS strain, establishing four replicate cross vials per UAS. Sixteen Gal4-UAS females were tested from each vial, a total of 64 per genotype, following the phenotyping pipeline described above (see *Phenotyping pipeline* and File S1). In addition, a group of 20 Gal4-UAS females from each genotype was maintained on test media lacking boric acid for the duration of the assay. None of these Gal4-UAS animals died by the end of the exposure assay, indicating that RNAi-based knockdown of the two target genes does not lead to gross effects on viability in the absence of boric acid.

### Xenobiotic metabolism genes in D. melanogaster

Five broad classes of genes are thought to be principally responsible for xenobiotic metabolism in eukaryotes. To determine which, if any, of these genes are present within QTL mapped in this study, we extracted from FlyBase (Attrill *et al.* 2016) all genes associated with the following Interpro IDs (Mitchell *et al.* 2015): cytochrome P450s (IPR001128), glutathione S-transferases (IPR004045, IPR004046, IPR010987), UDP-glucuronosyltransferases (IPR002213), esterases (IPR002018), and ABC transporters (IPR003439).

### Data availability

All individual-level phenotypes are provided in File S1. Raw activity monitor datafiles, along with R scripts to generate individual- and strain-level phenotypes for the DSPR, have been uploaded as a Dryad data package (doi: 10.5061/dryad.3v55m). DSPR QTL mapping output, including positions and LOD scores, is provided in File S2. Lists of genes currently annotated within mapped DSPR QTL are provided in File S3. Positions and association statistics for all nominally significant (*P* < 0.05) variants tested in the DGRP GWAS are provided in File S4. DSPR RILs can be requested from FlyRILs.org. 

## Results and Discussion

### Substantial trait variation and heritability

We measured the lifespan of mated female flies on 1.5% boric acid-supplemented media for 835 pA and 855 pB DSPR RILs, and 163 DGRP strains, testing 8 and 16 flies per genotype in the DSPR and DGRP, respectively (File S1). Figure 1 highlights the considerable among-genotype variation in boric acid resistance observed in all mapping panels. The three sets of genotypes exhibit similar average line phenotypes (41.3–43.6 hr), have comparable phenotypic ranges (DSPR pA, 11.3–91.3 hr; DSPR pB, 18.5–94.9 hr; and DGRP, 20.2–82.9 hr), and yield similar estimates of broad-sense heritability (DSPR pA = 47.0%; DSPR pB = 49.7%; and DGRP = 52.5%). Given that all replicate individuals tested for a single genotype were routinely derived from the same culture vial, and because every genotype was tested in just one experimental batch, some fraction of the variation we attribute to genetic factors is likely to result from environmental effects.

**Figure 2:**
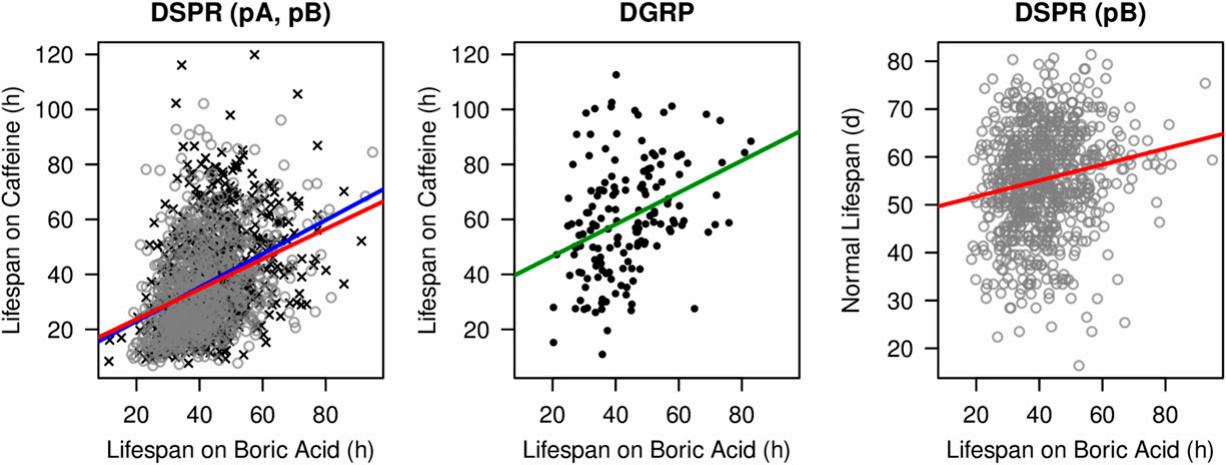
Phenotypic correlations in the DSPR and DGRP. Boric acid resistance data are from the current study, resistance to caffeine is from Najarro *et al.* (2015), and lifespan data under no-drug conditions (available for DSPR pB only) is from Highfill *et al.* (2016). Each point represents the mean phenotype of a single line from the DSPR pA (black cross), DSPR pB (gray open circle), and DGRP (black filled circle) populations. Only those strains with data from all relevant phenotypes are shown (822 DSPR pA RILs, 796 DSPR pB RILs, and 154 DGRP lines). Correlations between phenotypes are shown with colored lines: DSPR pA (blue), DSPR pB (red), and DGRP (green). Boric acid and caffeine resistance are highly correlated in both the DSPR (*r* = 0.37, *P* < 10^−15^) and DGRP (*r* = 0.36, *P* < 10^−5^). Boric acid resistance shows a more modest relationship with normal lifespan in DSPR pB RILs (*r* = 0.17, *P* < 10^−6^). DGRP, *Drosophila* Genetic Reference Panel; DSPR, *Drosophila* Synthetic Population Resource; RILs, Recombinant Inbred Lines.

We previously measured adult, mated female resistance to 1% caffeine in the DSPR and DGRP using a phenotyping pipeline identical to that employed in the current study (Najarro *et al.* 2015). Using those strains assayed for both phenotypes we find strong and significant positive correlations between phenotypes in all three panels of lines (Figure 2): DSPR pA (*n* = 822, Pearson’s *r* = 0.37, *P* < 10^−15^), DSPR pB (*n* = 796, *r* = 0.37, *P* < 10^−15^), and DGRP (*n* = 154, *r* = 0.36, *P* < 10^−5^). This result suggests that some fraction of the genetic variation contributing to resistance might be shared by these traits.

Highfill *et al.* (2016) measured the lifespan of mated females on regular, no-drug food in the DSPR pB panel. The correlation between lifespan and boric acid resistance is significant, although the magnitude of the relationship is relatively small (Figure 2, n = 796, *r* = 0.17, *P* < 10^−6^), and the phenotypic correlation between lifespan and caffeine resistance is low (*n* = 796, *r* = 0.07, *P* = 0.04). Calculating the correlation between the two measures of resistance while controlling for lifespan, using the *pcor* function in the R package *ppcor* (Kim 2015), reveals that the strong correlation between the resistance traits is maintained (*n* = 796, *r* = 0.36, *P* < 10^−24^). These results imply that variants contributing to overall longevity may generally not play a role in lifespan under drug-exposure conditions. Nonetheless, we note that the assay used to measure lifespan had little in common with that used to assay drug resistance, and these technical differences could lead to the underestimation of any correlation between traits.

### Multiple modest-effect boric acid toxicity QTL

**Figure 3:**
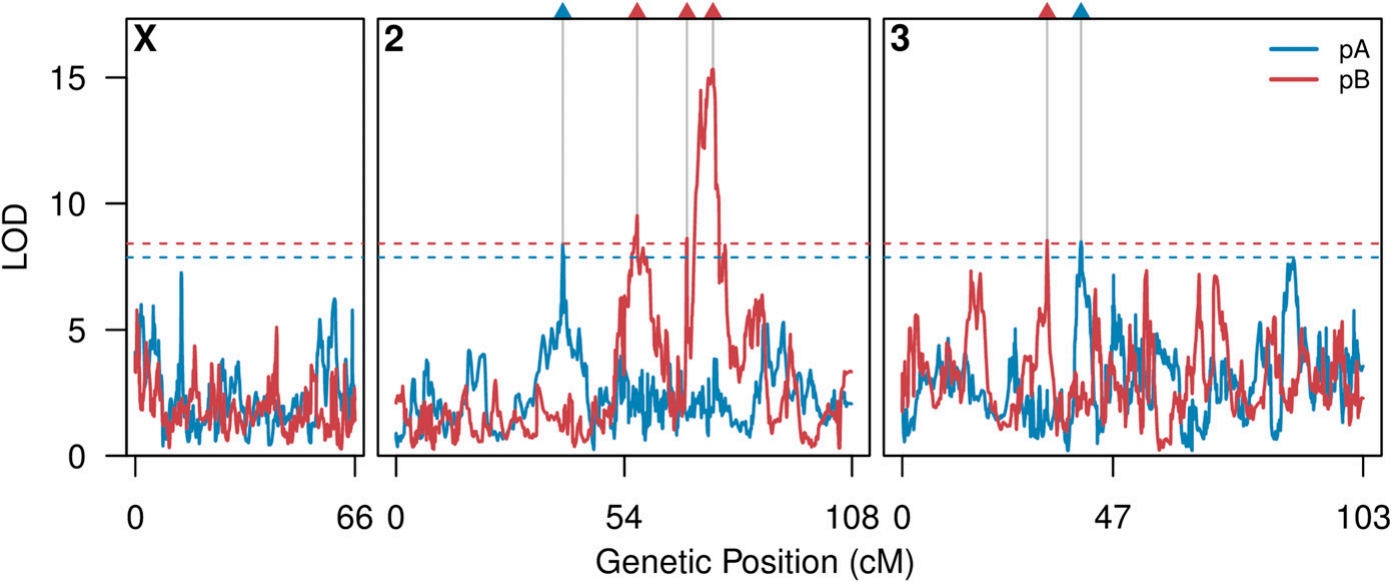
Genome-wide scan for boric acid resistance QTL in the DSPR. Solid lines depict population-specific scans across the major *D. melanogaster* chromosomes. Horizontal dashed lines represent permutation-derived 5% critical thresholds (pA, 7.9; pB, 8.4). Autosomal centromeres are shown at positions 54 cM (chromosome 2) and 47 cM (chromosome 3). The locations of the six mapped QTL are highlighted along the top of the plot, with colors indicating population (left-to-right; pA.Q1, pB.Q1, pB.Q2, pB.Q3, pB.Q4, and pA.Q2). DSPR, *Drosophila* Synthetic Population Resource; QTL, quantitative trait loci.

We mapped six, mapping panel-specific QTL in the DSPR (Figure 3, File S2, and Table 1). One QTL, pB.Q3, explains 7.9% of the phenotypic variation, while the remaining QTL each explain 4.5–5.0% of the variance. Assuming the QTL are independent and act additively, mapped QTL explain 9.1 and 21.9% of the variation in boric acid resistance in the pA and pB panels, respectively. Given that the power to map a 5% QTL in the DSPR with 800 RILs is 84% (King *et al.* 2012a), we reasoned that those genetic variants contributing to the remainder of the heritability are likely to be numerous and confer relatively small effects on phenotypic variance.

**Table 1:** Boric acid QTL mapped in the DSPR

QTL Name	LOD Score	Chr	Interval (cM)*^a^*	Interval (Mb)*^a^*	Genes*^b^*	Var Expl*^c^*
pA.Q1	8.4	2L	39.1–39.7	10.07–10.21	14 (1)	4.5
pA.Q2	8.5	3L	39.0–40.8	12.86–13.89	83 (42)	4.6
pB.Q1	9.5	2R	55.7–57.3	2.66–3.85	188 (32)	5.0
pB.Q2*^d^*	8.6	2R	68.6–68.8	9.56–9.67	5 (1)	4.5
pB.Q3*^d^*	15.3	2R	73.2–75.5	11.00–11.57	63 (11)	7.9
pB.Q4	8.5	3L	32.1–32.7	10.29–10.45	3 (8)	4.5

QTL, quantitative trait locus; LOD, logarithm of the odds; Chr, chromosome; Var Expl, variation explained.

a The 2-LOD support interval of the QTL. Physical positions (in Mb) are based on release 5 of the *D. melanogaster* reference genome.

b The number of protein-coding genes within the 2-LOD support interval, and (in parentheses) the number of other genes (*e.g.*, miRNA genes). Data were harvested from FlyBase (Attrill *et al.* 2016) after converting QTL positions from release 5 (reported by *DSPRqtl*) to release 6 coordinates.

c The proportion of the phenotypic variance due to each QTL. This value comes directly from the linear model used for mapping (Broman and Sen 2009, page 77).

d These QTL overlap with the positions of caffeine resistance QTL previously identified in the pB panel of the DSPR. pB.Q2 and pB.Q3 mapped in the current study overlap with Q4 and Q5, respectively, as reported in Table 1 from Najarro *et al.* (2015).

There is no overlap in the QTL identified in the pA and pB populations. Indeed, in regions harboring QTL in one panel, LOD curves in the other panel are not close to the genome-wide threshold, showing no signature of corresponding allelic effects (Figure 3). Similarly, Najarro *et al.* (2015) found that 7/10 caffeine resistance QTL were mapped in just one DSPR population. Absent power concerns, and given there is similar trait heritability in both DSPR panels (above), these results imply there is heterogeneity in the trait genetic architecture between DSPR mapping panels. This is perhaps unsurprising since panels were initiated from small, largely nonoverlapping sets of founding lines (just one founder is shared between populations). Causative alleles present in one panel may be absent in the other, or the alleles may be present in both but at very different frequencies, affecting mapping power, and/or the alleles may have background-specific effects. Without identifying the precise causative sites it will be difficult to ascertain why the DSPR panels reveal different sets of QTL.

**Figure 4:**
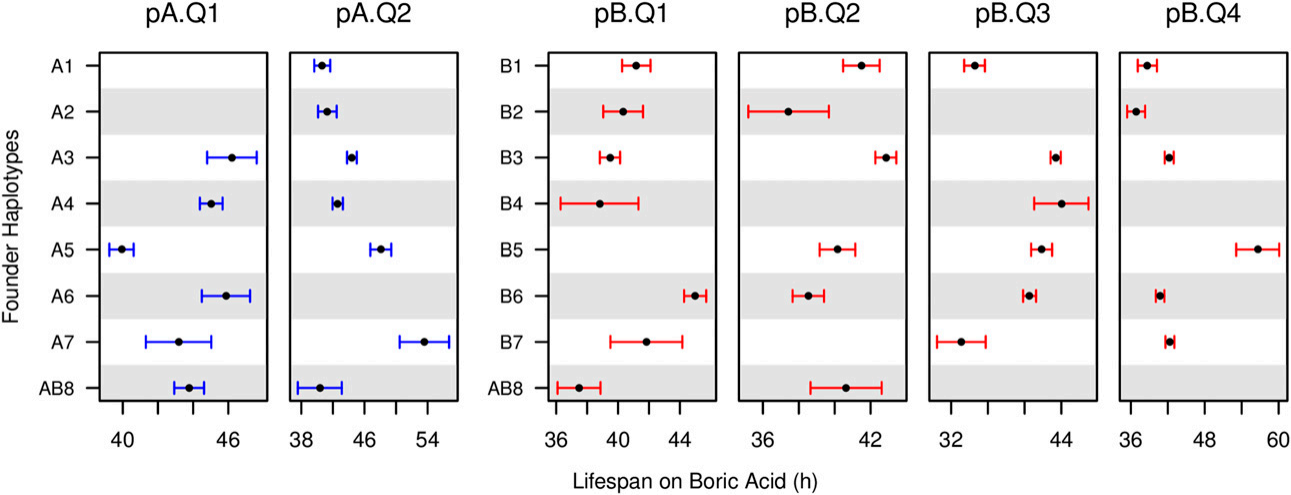
Founder haplotype means at mapped boric acid resistance QTL. RILs can harbor any of the eight possible founder haplotypes at each position. Averaging the phenotypes of RILs carrying each founder haplotype describes their contribution to a QTL. Founder haplotype means (filled circles) and 1-SE (whiskers) are calculated using only RILs for which we confidently assign a founder genotype at the position (probability > 0.95). Means are plotted only when based on at least 10 such observations; Certain haplotypes can be nearly absent at particular loci due to drift and/or selection occurring during the maintenance of the synthetic populations that led to the DSPR (King *et al.* 2012b). DSPR, *Drosophila* Synthetic Population Resource; QTL, quantitative trait loci; RILs, Recombinant Inbred Lines.

A feature of MPP approaches is that one can estimate the phenotypic effects of each founder haplotype at mapped QTL. The estimated effects at the six boric acid QTL do not clearly suggest that the QTL are each the result of a single biallelic variant (Figure 4). This observation is consistent with data from previous studies in the DSPR for a number of traits (Kislukhin *et al.* 2013; King *et al.* 2014a,b) and has been seen in studies using other MPPs, such as the mouse DO population (Gu *et al.* 2016), the rat NIH-HS population (Rat Genome Sequencing and Mapping Consortium *et al.* 2013), and in maize (Giraud *et al.* 2014). The strain effects shown in Figure 4 could result from the action of multiple, tightly-linked QTL in each region, or from allelic heterogeneity, where multiple functional alleles segregate at single causative genes, as is routinely observed in human Mendelian disease genes. The strain effects will be valuable for elucidating the causative loci in the future. For example, one might expect to see a correlation between the founder mean phenotypes at a QTL, and the level of expression of a causative gene in a relevant tissue among founders.

Three of the QTL (pA.Q1, pB.Q2, and pB.Q4) are mapped to small genetic (0.27–0.61 cM) and physical (110–140 kb) intervals, and harbor 3–14 protein-coding genes (File S3 and Table 1). The interval for pA.Q1 harbors the gene *Sur* (*Sulfonylurea receptor*, FBgn0028675), which encodes an ABC transporter. This locus represents a strong candidate to carry functional variation impacting resistance to boric acid, since transporters are required to excrete the products of xenobiotic metabolism from cells (Xu *et al.* 2005). Notably, proper expression of *Sur* has been shown to be required for heart function (Akasaka *et al.* 2006), and heart-specific RNAi knockdown of *Sur* leads to reduced survival following injection with the flock house RNA virus (Croker *et al.* 2007). One of five protein-coding genes implicated by the pB.Q2 interval is *fas* (*faint sausage*, FBgn0000633), which when mutated alters the morphology of malpighian tubules (Jack and Myette 1999; Liu *et al.* 1999), structures critical for xenobiotic metabolism (Dow and Davies 2006; Yang *et al.* 2007). QTL pB.Q4 harbors a pair of ionotropic receptors, *Ir67b* (FBgn0036083) and *Ir67c* (FBgn0052058), a gene family involved in chemosensation (Benton *et al.* 2009; Stewart *et al.* 2015) but lacking any clear link to xenobiotic detoxification. However, the phenotyping assay we employed does not involve a check on the amount of media ingested by test animals. Our screen could therefore result in QTL that contribute to the propensity to ingest a toxic substance, and variation in chemosensory genes would be strong candidates to underlie such QTL.

The remaining three QTL (pA.Q2, pB.Q1, and pB.Q3) are more broadly mapped, implicating 63–188 protein-coding genes (File S3 and Table 1), making it more difficult to hypothesize plausible candidates for follow-up, functional testing. However, a search for members of those gene families known to be involved in xenobiotic metabolism (see *Materials and Methods*) revealed four P450s among the 188 protein-coding genes residing within the pB.Q1 interval; *Cyp6a2* (FBgn0000473), *Cyp6u1* (FBgn0033121), *Cyp9b1* (FBgn0015038), and *Cyp9b2* (FBgn0015039). Expression of *Cyp6a2* has been positively associated with resistance to the insecticide DDT (Wan *et al.* 2014), but other than being members of the P450 family no specific evidence links the other three genes to roles in xenobiotic resistance (Attrill *et al.* 2016). Chung *et al.* (2009) used *in situ* hybridization against the majority of *D. melanogaster* P450s, finding expression of *Cyp6a2* and *Cyp9b2* in the third instar larval midgut and malpighian tubules. *Cyp6u1* was expressed in gonadal tissue rather than in the gut, while no expression of *Cyp9b1* was detected. Thus, *Cyp6a2* and *Cyp9b2* represent the strongest candidates within pB.Q1 to contribute to boric acid resistance.

### Cyp9b2 RNAi reduces boric acid resistance

**Figure 5:**
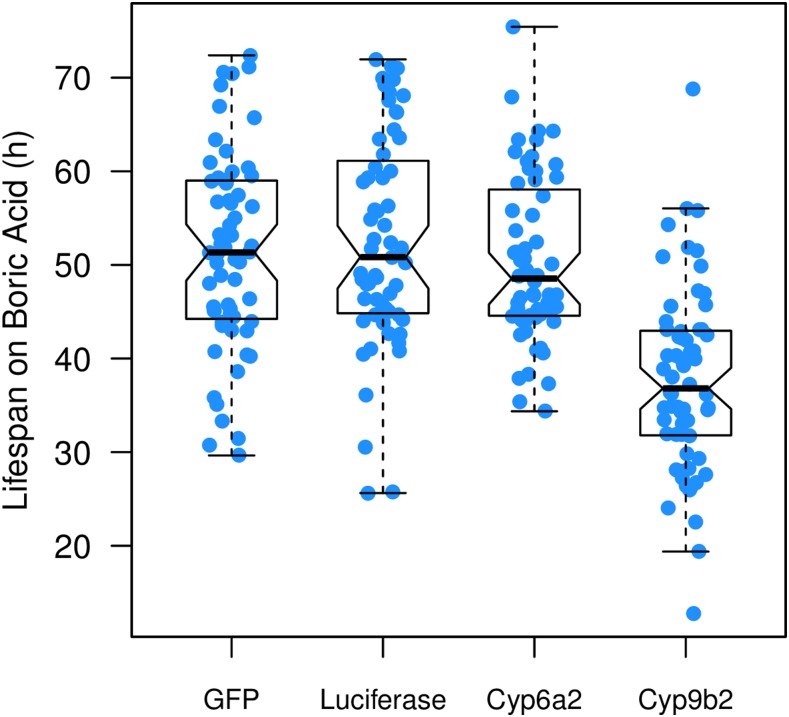
RNAi knockdown of candidate cytochrome P450 genes. We expressed Gal4 in all cells at all timepoints under the control of the *Actin 5C* promoter, driving GFP and luciferase as controls, and knocking down expression of the genes *Cyp6a2* and *Cyp9b2* (both residing under DSPR QTL pB.Q1). Raw phenotypes (59–64 animals per genotype) are presented as blue filled circles, along with a standard boxplot (generated using the R *boxplot* command). The *Cyp9b2* knockdown genotype shows a significant reduction in resistance compared to all other genotypes (Welch’s *t*-tests, *P* < 10^−11^). All other genotype pairs are not significantly different (*P* > 0.2). DSPR, *Drosophila* Synthetic Population Resource; GFP, green fluorescent protein; QTL, quantitative trait loci; RNAi, RNA interference.

To test for any functional effects of genes *Cyp6a2* and *Cyp9b2* on boric acid resistance we employed RNAi, knocking down each gene ubiquitously, and comparing to control genotypes expressing the markers GFP or luciferase (Figure 5). Knockdown of *Cyp6a2* yielded no change in phenotype compared to controls (Welch’s *t*-tests, *P* > 0.2), whereas reduction in *Cyp9b2* expression markedly lowered resistance to boric acid (*P* < 10^−11^), suggesting that *Cyp9b2* is functionally important for resistance.

Clearly, experimental knockdown of gene expression in all cells and at all timepoints may not reflect the effect of any natural *Cyp9b2* allele segregating within the DSPR, and positive RNAi knockdown is not strong evidence for the existence of such allelic variation. Preadult knockdown of *Cyp9b2* could additionally negatively impact adult fitness, indirectly resulting in reduced lifespan during drug exposure. Furthermore, RNAi can lead to nonspecific effects, affecting the expression of genes other than the target (Ma *et al.* 2006), potentially falsely implicating a given gene in the control of a phenotype. Notwithstanding these caveats, *Cyp9b2* represents a good target for future analyses seeking to identify causative variants impacting boric acid resistance. We note that we cannot rule out the possibility that genes other than *Cyp9b2* could additionally be responsible for the QTL effect observed, particularly since pB.Q1 is relatively broad (Table 1).

### Overlap among xenobiotic resistance QTL in the DSPR

Two of the six boric acid QTL, pB.Q2 and pB.Q3, overlap with QTL contributing to variation in caffeine resistance that we previously mapped in the DSPR pB panel (Table 1 in Najarro *et al.* 2015). Given the relatively short intervals implicated by mapped QTL, the nearly identical phenotyping pipelines employed in each study, and the phenotypic correlations observed between the two traits (Figure 2), it is conceivable that resistance to both drugs is associated with the same loci in these intervals. A correlation between the strain effects estimated at each overlapping pair of QTL would provide additional support for this contention. However, while the correlations are positive and high, since only 6/8 founders are present at these locations (see Figure 4), they are not significant (Pearson’s *r* = 0.53–0.70, *P* > 0.12 in each case).

Assuming the causative variants are pleiotropic, since boric acid QTL pB.Q2 resides completely within caffeine resistance QTL Q4, this would reduce the number of protein-coding genes implicated in this latter QTL from 26 (see Najarro *et al.* 2015) to 5 (File S3 and Table 1). The intervals for boric acid QTL pB.Q3 and caffeine QTL Q5 also overlap, and if the QTL have the same genetic cause the size of the region containing any pleiotropic variant(s) would be reduced from 570 kb (boric acid pB.Q3, Table 1) and 650 kb (caffeine QTL Q5) to 460 kb, a region containing 47 protein-coding genes.

Simple overlap of QTL does not provide unambiguous evidence for pleiotropy, particularly when each QTL implicates multiple genes as we see here. Overlap may be purely stochastic, with different, very closely-linked loci within the overlapping region contributing independent effects to the different phenotypes. Functional tests of plausible candidate genes within overlapping QTL regions can directly address whether some of the genetic control of resistance to boric acid and caffeine is shared.

### GWAS for boric acid resistance loci

MPP mapping designs have considerable power to detect relatively short regions of the genome containing alleles influencing complex trait variation (Valdar *et al.* 2006a; King *et al.* 2012a; Gatti *et al.* 2014). This power derives from the fact that in an MPP one is testing the effect of a local haplotype on phenotype, in effect integrating over any and all causative variants in that region in a single test. A caveat with this approach is that mapping resolution is necessarily diminished relative to a population-based association study. In such a study LD (linkage disequilibrium) likely extends a relatively short distance, and any associated variant is likely to be the causative site, or at worst be very close to the causative site (Risch and Merikangas 1996). In the DSPR, within fragments of the genome not subject to crossing over, many variants are in perfect LD, and all tag the exact same haplotype(s). This phenomenon can be seen in QTL-specific association scans. In these analyses, we use the variants identified in the founders together with the estimated mosaic haplotype structure of the RILs (King *et al.* 2012b) to infer the alleles present in each RIL, and carry out a series of single marker association tests. Figure S1 shows that many SNPs within each QTL interval are strongly associated with phenotype, and while these tests might allow variants to be ranked, they do not confidently point to highly likely candidate causative sites.

**Figure 6:**
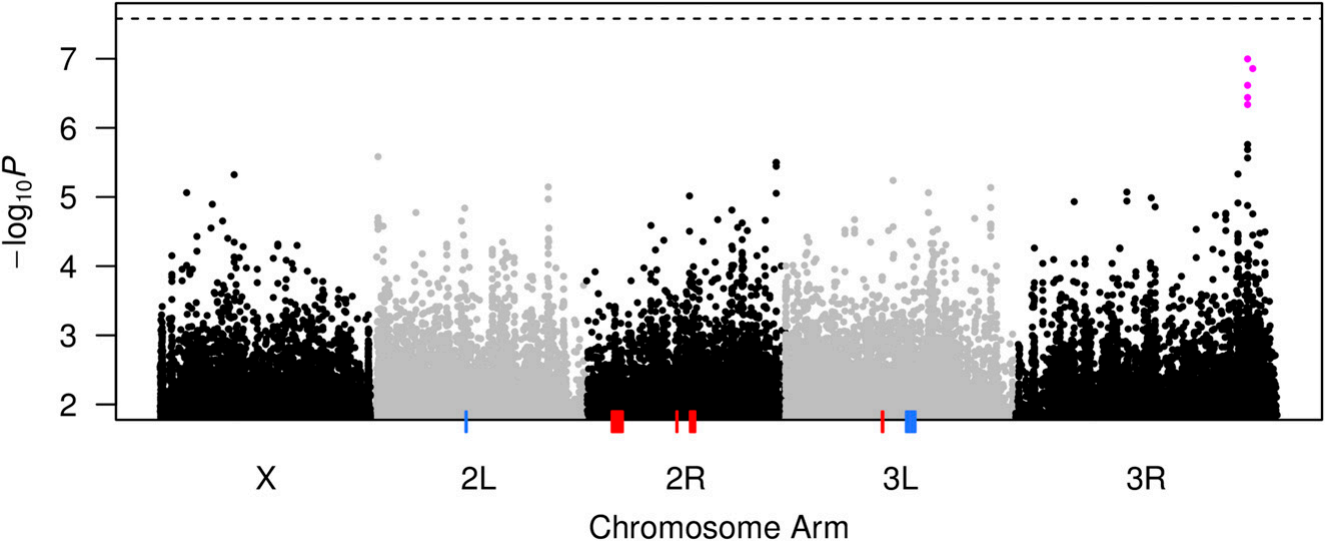
Manhattan plot of DGRP-based boric acid resistance GWAS. Association testing was carried out at 1.9 million common variants, accounting for variation across strains in relatedness, *Wolbachia* infection status, and chromosomal inversion genotype. The strength of each association is presented as a −log_10_(*P*-value) with variants on the major chromosome arms delineated by alternating black/gray symbols. The set of five associations toward the right end of chromosome 3R (magenta symbols) survive a genome-wide 20% FDR threshold. The horizontal dashed line at the top of the plot represents a 5% genome-wide Bonferroni correction for multiple tests (*P* = 2.6 × 10^−8^). The blue/red colored rectangles along the *x*-axis represent the physical positions of the six QTL mapped in the DSPR (left-to-right; pA.Q1, pB.Q1, pB.Q2, pB.Q3, pB.Q4, and pA.Q2). DGRP, *Drosophila* Genetic Reference Panel; DSPR, *Drosophila* Synthetic Population Resource; FDR, False Discovery Rate; GWAS, genome-wide association study; QTL, quantitative trait loci.

To attempt to resolve nucleotide sites contributing to variation in boric acid resistance we turned to the DGRP (Mackay *et al.* 2012; Huang *et al.* 2014), a series of inbred strains of *D. melanogaster* derived from a single collection site, and subjected to short-read sequencing. The distribution of phenotypes across the 163 strains assayed, and the heritability of the phenotype in the DGRP is comparable to that seen in the DSPR (Figure 1). Following GWAS analysis, no variant survives a conservative genome-wide 5% Bonferroni multiple testing threshold (Figure 6, horizontal dashed line).

Identifying no or very few variants surviving a strict Bonferroni threshold is common in GWAS studies employing the DGRP (*e.g.*, Arya *et al.* 2015; Dembeck *et al.* 2015; Carbone *et al.* 2016; Akhund-Zade *et al.* 2017), and such analyses commonly report interesting associations as those surviving an arbitrary threshold of *P* < 10^−5^. In the present study, this threshold corresponds to an FDR of 79% (File S4), likely providing limited value in elucidating causative genes/variants. Instead, we explored several more stringent FDR thresholds, identifying five variants toward the end of chromosome 3R that survive a genome-wide 20% FDR threshold (Figure 6, magenta symbols; File S4). These five are the only variants that survive a genome-wide 50% FDR threshold, and none are present within those QTL intervals implicated by the DSPR (Figure 6).

One of the 20% FDR variants (3R:24708824 in release 5 of the *D. melanogaster* reference genome) is present within an intron of gene *CG33203* (FBgn0053203), for which there is limited information on FlyBase (Attrill *et al.* 2016). Approximately 5–7 kb downstream is a cluster of three variants (3R:24714040, 3R:24716025, and 3R:24716095) residing within *Doa* (*Darkener of apricot*, FBgn0265998). This locus encodes a serine/threonine protein kinase, and structural mutations in the gene increase resistance to paraquat-induced oxidative stress (James *et al.* 2009). These four variants are present within a 7 kb section of the genome, show relatively high levels of LD (*r*^2^ = 0.55–0.88), and thus may all be proxies for a single causative variant. The fifth associated variant is > 500 kb further downstream (3R: 25288112), shows limited LD with the four upstream variants (*r*^2^ = 0.07–0.12), and is present within the *Ptp99A* gene (FBgn0004369). Ubiquitous RNAi of this protein tyrosine phosphatase has been shown to exhibit increased levels of tolerance to ethanol and benzyl alcohol (Ghezzi *et al.* 2013).

Validation of DGRP “hits” (meaning sites with association statistics significant at *P* < 10^−5^) via RNAi of genes containing those hits, or analysis of mutations in such genes, is frequently quite successful. This suggests that the sets of genes containing sites yielding small *P*-values in the GWAS may be enriched for those with true effects on phenotype. For instance, four recent DGRP studies showed that 64–88% of genes containing hits that were functionally tested showed a significant effect on phenotype (Arya *et al.* 2015; Carbone *et al.* 2016; Vonesch *et al.* 2016; Akhund-Zade *et al.* 2017). Notably, Vonesch *et al.* (2016) additionally showed that the rate of functional validation for GWAS hits was significantly higher than that for a set of random genes, again suggesting that despite the modest level of statistical support for DGRP-based GWAS hits, as a class they appear to be enriched for true positives. Based on these results, future functional testing targeted at *Doa* and *Ptp99A* may help to confirm any effects of these loci on boric acid resistance.

### Limited overlap between loci implicated by the DSPR and DGRP

A key question is whether there is any signature of associated variants in the DGRP within DSPR-derived QTL intervals, since these would help to implicate nucleotide sites in the control of trait variation. We extracted association tests carried out at variants within each QTL region, and explored various QTL-specific multiple testing thresholds. No variants survive QTL-specific 5% Bonferroni or 10% FDR thresholds. At 20% FDR, 5/6 QTL still show no associated variants, but 4/4415 variants tested within pB.Q4 do survive. All four of these sites are within 90 bases of each other (3L:10341876–10341966) in *CR46006* (FBgn0267668), one of the 11 genes residing within the pB.Q4 interval. No information is currently available regarding the function of this nonprotein-coding gene (Attrill *et al.* 2016), but given the precision with which pB.Q4 is mapped in the DSPR (Table 1), and the presence of these suggestive associations in the DGRP, future testing of the effects of this gene on boric acid resistance are warranted.

Ultimately, the level of overlap between the DSPR and DGRP is disappointingly low, consistent with our previous work on caffeine resistance (Najarro *et al.* 2015). Combining results over the two panels does not strongly improve the ability to identify causative nucleotide sites underlying trait variation. Part of the difficulty is certainly the low power in the DGRP (Turner *et al.* 2013; Long *et al.* 2014). With 163 lines, the power to identify a common (50% minor allele frequency) variant contributing 5% to the total phenotypic variation, which broadly corresponds to the estimated effects of our mapped QTL (Table 1), is ∼6% at a genome-wide 5% Bonferroni threshold (based on testing close to 1.9 million sites). QTL intervals contain 2615–18754 tested variants, and at a more liberal threshold of *P* < 10^−5^ appropriate for a QTL-centric association study, power remains at a relatively low ∼39%. Power will be lower still if DSPR QTL are multiallelic, as implied by Figure 4 and by a range of MPP studies indicating that the loci contributing to complex traits are commonly multiallelic (Kislukhin *et al.* 2013; Rat Genome Sequencing and Mapping Consortium *et al.* 2013; Giraud *et al.* 2014; King *et al.* 2014a,b; Gu *et al.* 2016). Multiallelic QTL are the result of the action of many, likely rare alleles, with independent small effects on phenotype that will be very difficult to detect individually using a traditional GWAS framework (Pritchard 2001; Thornton *et al.* 2013).

A further concern with the GWAS approach is that one can only detect the effects of variants that are directly genotyped, or those that are in strong LD with a genotyped variant. Any other variants are effectively invisible to the approach. This is in contrast to MPP-based mapping, where methods test for the effects of short haplotypes on phenotype and are agnostic as to the molecular nature of the causative variants. In this respect, DGRP genotypes are currently based on short-read sequencing data, which is likely to miss a range of structural variants (Pendleton *et al.* 2015). Copy number variants and transposable element insertions have been previously implicated in xenobiotic resistance (*e.g.*, Daborn *et al.* 2002; Chung *et al.* 2007; Schmidt *et al.* 2010). So, to the extent that this class of variants are important contributors to boric acid and caffeine resistance, are absent from the DGRP variant catalog, and are not “tagged” by genotyped sites due to the very low LD in the DGRP (Mackay *et al.* 2012), and in *D. melanogaster* more generally (*e.g.*, Macdonald *et al.* 2005), the ability to find resistance loci in the DGRP may be undermined.

Finally, despite the similar trait distributions (Figure 1), the genetic architecture of variation in boric acid resistance may show heterogeneity between the DGRP and DSPR populations. The DSPR is constructed from a worldwide collection of founding strains that lack *P* transposable elements, and that have been maintained for decades in labs since their collection (King *et al.* 2012b), whereas the DGRP is constructed from lines harboring *P*-elements derived more recently from a single location in North America (Mackay *et al.* 2012). We might expect such populations to differ in the genetic control of trait variation. Indeed, hundreds of thousands of SNPs are unique to one of the two mapping populations (Najarro *et al.* 2015; King and Long 2017), so any phenotypic effects they confer cannot be replicated across mapping populations. Even for those variants that are shared between the DSPR and DGRP, allele frequencies are not strongly correlated between the panels (King and Long 2017). Thus, even if a causative variant is present in both the DSPR and DGRP, different allele frequencies could markedly impact the power to find it in each panel. For instance, GWAS methods are highly underpowered to detect very rare causative alleles (Pritchard 2001), so a causative variant that is truly rare in the population but was captured in one of the DSPR founders and led to a mapped QTL would be hard to replicate in the DGRP. Finally, shared variants may be background-dependent, conferring detectable effects on phenotype only in one of the two mapping panels. Epistasis as an explanation for the failure to replicate variants contributing to complex trait variation across mapping populations has been proposed previously (Huang *et al.* 2012), although other phenomena, such as mapping power and Beavis effects (King and Long 2017), are likely important contributors to such lack of validation across mapping panels.

There is an array of biological and technical factors that could lead to differences between the population-based DGRP GWAS approach and the MPP-based DSPR linkage mapping approach. However, a principal difference is that the strains from which the populations are derived are distinct. Deriving recombinant MPP mapping populations from subsets of the DGRP strains can control for the difference in mapping methodology, may allow a more effective contrast between the DGRP and DSPR genotypes, and permit an exploration of the heterogeneity in trait architecture across populations.

### Conclusions

In this study, we mapped several loci that impact resistance to the xenobiotic boric acid, resolving these modest-effect QTL to regions implicating relatively small numbers of genes. We highlight plausible candidate genes for some mapped QTL that can be targets of future studies to elucidate the mechanisms of boric acid toxicity in insects. Perhaps surprisingly, only two of the QTL implicate members of gene families known to be responsible for metabolism of xenobiotics: one QTL contains an ABC transporter (*Sur*) and a second contains multiple P450s. We confirm that one of these P450s, *Cyp9b2*, affects boric acid resistance using RNAi knockdown, although we recognize that while RNAi is a useful and efficient tool to implicate a gene in the control of a phenotype, a positive result is a weak standard of evidence that the gene harbors variants that contribute to natural trait variation. It is possible that those QTL lacking recognized detoxification genes are generated by variants that lead to morphological or behavioral differences across strains that impact xenobiotic resistance indirectly, rather than by directly affecting boric acid metabolism. A benefit of large, inbred reference panels is that one can pursue more detailed phenotypic characterization of phenotypically-extreme strains, collect genomics data at a range of levels (*e.g.*, transcriptomics and metabolomics), use a systems genetics approach to generate a more granular picture of phenotypic variation, and help identify the variants that produce it.

Our attempt to marry DSPR-based QTL mapping with a DGRP-based association study to identify putative causative variants within QTL was largely unsuccessful, as it was in our previous effort (Najarro *et al.* 2015). While the approach has appeal—broadly map a QTL in the DSPR and fine map that QTL to the nucleotide level with the DGRP—its success is predicated both on the genetic architecture of a trait being fairly consistent across mapping panels, and on having power in both panels to detect genes segregating for alleles with fairly subtle effects on phenotype that may be at relatively low frequency. At the level of both SNP allelic variation and transposable elements, the DGRP and DSPR show modest overlap (Cridland *et al.* 2013; King and Long 2017), suggesting genetic architecture for a trait may often not be the same over panels. In addition, straightforward power calculations (Long *et al.* 2014) demonstrate that QTL-centric association scans employing 200 DGRP strains will typically be insufficient to identify intermediate (or lower) frequency alleles with small effects on phenotype. As shown by a wealth of human genetics work, much larger and therefore more powerful association studies employing thousands of genotypes are necessary to pinpoint causative loci. And if the causative variants, their allele frequencies, or their effects on phenotype are not recapitulated in different populations, fully elucidating trait architecture will entail mapping in a number of separate populations.

## Supplementary Material

Supplemental material is available online at www.g3journal.org/lookup/suppl/doi:10.1534/g3.117.041418/-/DC1.
