# Supplementary material for: Loci Contributing to Boric Acid Toxicity in Two Reference Populations of Drosophila melanogaster
Source: G3 (Bethesda). 2017 Jun 5;7(6):1631–41. doi: 10.1534/g3.117.041418 (PMC5473745; doi:10.1534/g3.117.041418)

Figure S1 QTL-specific association scans. Each point is a variant segregating in the DSPR founders. SNP alleles in each RIL are inferred based on the estimated mosaic founder structure of each RIL (see KING *et al.* 2012b), and tested against phenotype using a single marker model. QTL positions are those indicated in Table 1. Variants in blue survive a region-specific 1% FDR threshold, while variants in red survive a region-specific 5% Bonferroni threshold. The clear "stripes" of points across each plot are due to the haplotypic structure of the DSPR RILs; In general, sites are in very tight LD with many other sites.

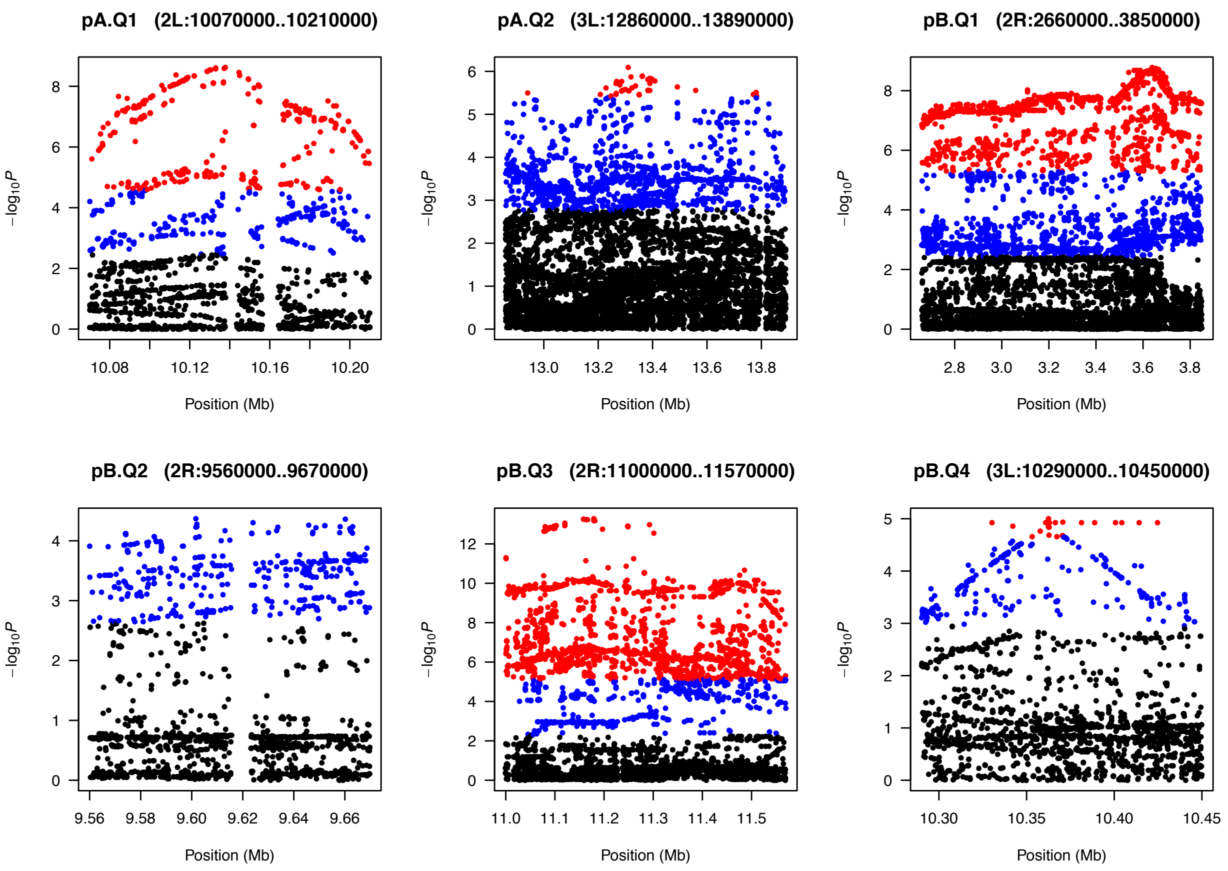

Supplement: Supplementary file 1 [file 1631File004.pdf]
