# Supplementary material for: Loci Contributing to Boric Acid Toxicity in Two Reference Populations of Drosophila melanogaster
Source: G3 (Bethesda). 2017 Jun 5;7(6):1631–41. doi: 10.1534/g3.117.041418 (PMC5473745; doi:10.1534/g3.117.041418)
Supplement: Supplementary file 2 [file 1631File006.pdf]

TITLE
Loci Contributing to Boric Acid Toxicity in Two Reference Populations of *Drosophila melanogaster*

AUTHORS
Michael A. Najarro, Jennifer L. Hackett, and Stuart J. Macdonald (sjmac@ku.edu)

Genetic material
DSPR strains are available from the Macdonald lab, and can be requested via our project website (FlyRILs.org). The strain naming scheme is described in the legend to supplementary File S1 given below. DGRP strains are available from the Bloomington *Drosophila* Stock Center (flystocks.bio.indiana.edu). Statements regarding strain availability are provided within the Materials and Methods section of the manuscript.

Raw genotype/marker information
Genotypes are available via our project website FlyRILs.org, and were uploaded to Dryad (dx.doi.org/10.5061/dryad.r5v40) as part of a previous DSPR publication in *Genetics* (Marriage et al. 2014; PMID: 25236448). A statement regarding genotype data availability is provided within the Materials and Methods section of the manuscript.

Raw phenotype data
All phenotypes collected as part of the study are available within supplementary File S1 (see legend below). Raw activity monitor datafiles, along with R scripts to generate individual- and strain-level phenotypes for the DSPR, have been uploaded as a Dryad data package (doi:10.5061/dryad.3v55m).

Descriptions of phenotypes
This information is given in the Materials and Methods section of the manuscript. We additionally cite a previous open-access paper from our group (Najarro et al. 2015; PMID: 26619284) that employs an essentially identical pipeline to assay a related phenotype, and provides even more detail on the methodology.

Results files
We provide three results files that will be included as supplementary files associated with the paper. File S2 provides genomewide LOD scores, and physical/genetic positions, for both panels of the DSPR MPP. File S3 provides lists of genes that are currently annotated within all mapped QTL. File S4 provides results of the DGRP GWAS, including physical positions and significance values. Legends for all files are given below.

Software
The DSPRqtl R-based analysis package is available from our project website (FlyRILs.org) and from GitHub (github.com/egking/DSPRqtl). Statements to this effect are provided in the Materials and Methods section of the manuscript. DGRP data was analyzed through the web-based DGRP2 analytical engine (dgrp2.gnets.ncsu.edu). Information about the analytical methodology employed in this analysis is given in Huang et al. (2014; PMID: 24714809).

Legends for supplementary datafiles

File S1 Phenotype of every individual scored in the study. A three-column tab-delimited text file. The "expt" column associates each individual with the DSPR QTL mapping study, the DGRP association study, or the RNAi experiment. The "genotype" column associates each individual with its genotype code. DSPR genotypes are given as 5-digit RIL codes. The first digit indicates population (1 = pA, 2 = pB), the second digit indicates subpopulation (1 = pA1 or pB1, 2 = pA2 or pB2), and the final three digits are subpopulation-specific RIL codes. DGRP genotypes are given as 5-digit Bloomington *Drosophila* Stock Center (BDSC) codes concatenated to the equivalent, and commonly used 'RAL number' genotype codes. For the RNAi study, genotypes are described by the cross used to produce them. All RNAi genotypes are the progeny of crossing males from BDSC stock 25374 (*Act5C*-GAL4) to females from four different UAS-transgene stocks: 35786 (UAS-*GFP*), 35788 (UAS-*Luciferase*), 62996 (UAS-*Cyp9b2-RNAi*), and 64008 (UAS-*Cyp6a2-RNAi*). The final component of the RNAi genotype name is the replicate cross vial from which each animal was harvested (replicates R1, R2, R3, and R4). The "phenotype" column is the lifespan, in hours, for the individual on media supplemented with 1.5% boric acid.

File S2 LOD scores from DSPR QTL mapping. A five-column tab-delimited text file. The "chr" column indicates the chromosome arm (X, 2L, 2R, 3L, 3R), the "pposR5" provides the physical position relative to Release 5 of the *Drosophila melanogaster* reference genome, and "gpos" gives the genetic position (in cM). The "LOD.pA" and "LOD.pB" columns give the LOD scores for the pA and pB populations, respectively. The 5% permutation-derived critical LOD thresholds used to call QTL are 7.86 (pA) and 8.41 (pB).

File S3 Genes residing within mapped DSPR QTL. A six-column tab-delimited text file. The "QTL" column indicates the mapped QTL, "cpos" indicates the cytological position of the gene, "pposR6" is the physical position of the gene relative to Release 6 of the *Drosophila melanogaster* reference genome, "GeneSymbol" and "FBgn" provide information on the gene name, and "Feature_Type" indicates the type of gene as assigned by FlyBase (ATTRILL *et al.* 2016).

File S4 Nominally-significant associations from DGRP GWAS. A five-column tab-delimited text file. The "chr" column indicates the chromosome arm (X, 2L, 2R, 3L, 3R), and the "pposR5" column provides the physical position of the tested variant relative to Release 5 of the *Drosophila melanogaster* reference genome. The "maf" column gives the minor allele frequency of the variant under test. The "pval" column provides the *P*-value of the association test using a linear mixed model, and is pulled directly from the "SingleMixedPval" column in the output of the DGRP2 analytical engine (dgrp2.gnets.ncsu.edu). The "fdr" column provides the FDR associated with each variant, generated using the entire set of 1.9 million test positions on the five major chromosome arms via the *p.adjust* R function. Only those variants with *P*-values below 0.05 are provided in the file.
